# Neuroprotective Potential of Free Radical-Scavenging Nanoparticles in Addressing Inflammation and Obesity

**DOI:** 10.1049/nbt2/6805313

**Published:** 2025-10-26

**Authors:** Shampa Ghosh, Rakesh Bhaskar, Krishna Kumar Singh, Bhuvaneshwar Yarlagadda, Prashant Verma, Sung Soo Han, Shiv Dutt Purohit, Jitendra Kumar Sinha

**Affiliations:** ^1^GloNeuro, Sector 107, Vishwakarma Road, Noida 201301, Uttar Pradesh, India; ^2^School of Chemical Engineering, Yeungnam University, Gyeongsan-si 38541, Republic of Korea; ^3^Symbiosis Centre for Information Technology (SCIT), Symbiosis International (Deemed University), Hinjawadi, Pune 411057, Maharashtra, India; ^4^BML Munjal University, NH8, Sidhrawali, Gurugram 122413, Haryana, India; ^5^Research and Innovation Cell, Rayat Bahra University, Mohali 140301, Punjab, India

**Keywords:** artificial intelligence, clinical perspectives, nanomedicine, neuroinflammation, therapeutic interventions, translational research

## Abstract

Neuroprotection is well known for its strategies and interventions that help preserve the structure and function of neurons during a myriad of neurological challenges. It is fundamental in managing the complex relationship between neuroinflammation and obesity, both of which are significant factors affecting our neurological health. In the present review, we try to merge nanoparticles with artificial intelligence (AI) to tackle the neurological implications of both conditions. This review summarizes prior studies of free radical-scavenging nanoparticles: polymeric, liposomal, ceria-based, and quantum dots, and evaluates their reported efficacy in attenuating markers of neuroinflammation and neuronal dysfunction in preclinical models. We have also discussed AI applications, such as predictive modeling and real-time monitoring, stating that they present a complementary role in themselves. There is recognition that the promise of nanoparticles in mitigating neurological problems underscores the potential of AI in upgrading neuroprotection. Early-phase clinical trials of free radical-scavenging nanoparticles have highlighted the importance of patient stratification to optimize personalized treatment regimens. Furthermore, we advocate coordinated efforts in education, awareness, and research to integrate scientific findings, public policy, and technology innovation, thereby holistically addressing neuroinflammation and obesity at the individual level.

## 1. Introduction

The interaction between neuroinflammation and obesity is convoluted but has emerged as a primary concern in recent research. It is capturing the interest of both the neuroscience and medical fields [1–3]. Neuroinflammation is usually linked to CNS infections or injuries and has become increasingly important in terms of chronic neurological illnesses [3]. Simultaneously, the increasing incidence of obesity, which is frequently associated with systemic inflammation, has drawn focus to the complex connection between metabolic health and brain function. These disorders also involve inflammatory mediators, oxidative stress, and immunological responses [4]. Neuroinflammation has been found to be involved in several neurological disorders, including Alzheimer's disease, Parkinson's disease, and multiple sclerosis [5–7]. Microglial activation and the subsequent release of cytokines lead to neuronal injury and dysfunction in the inflammatory cascade [8]. Nevertheless, obesity is characterized by insistent mild inflammation, where fatty tissues release proinflammatory cytokines that can affect brain activity and worsen neuroinflammation conditions [9, 10]. Comprehension and treatment of neuroinflammation and obesity are essential for neuroprotection, which refers to the safeguarding of neuronal structure and function [9, 11]. Neuroinflammation plays a significant role in the progression of neurodegenerative disorders, and methods for regulating inflammation have emerged as possible treatment approaches [5, 12]. Likewise, the inflammation caused by obesity can negatively affect cognitive well-being, perhaps leading to neurodegeneration and a loss of cognitive ability [11, 12]. The importance of tackling these challenges goes beyond personal well-being and has broader societal ramifications. The prevalence of neurodegenerative illnesses and cognitive decline is projected to increase due to the aging population and the global increase in obesity rates [13, 14]. Hence, novel strategies that can effectively address both neuroinflammation and obesity are urgently needed to achieve better neuroprotection.

**Figure 1:**
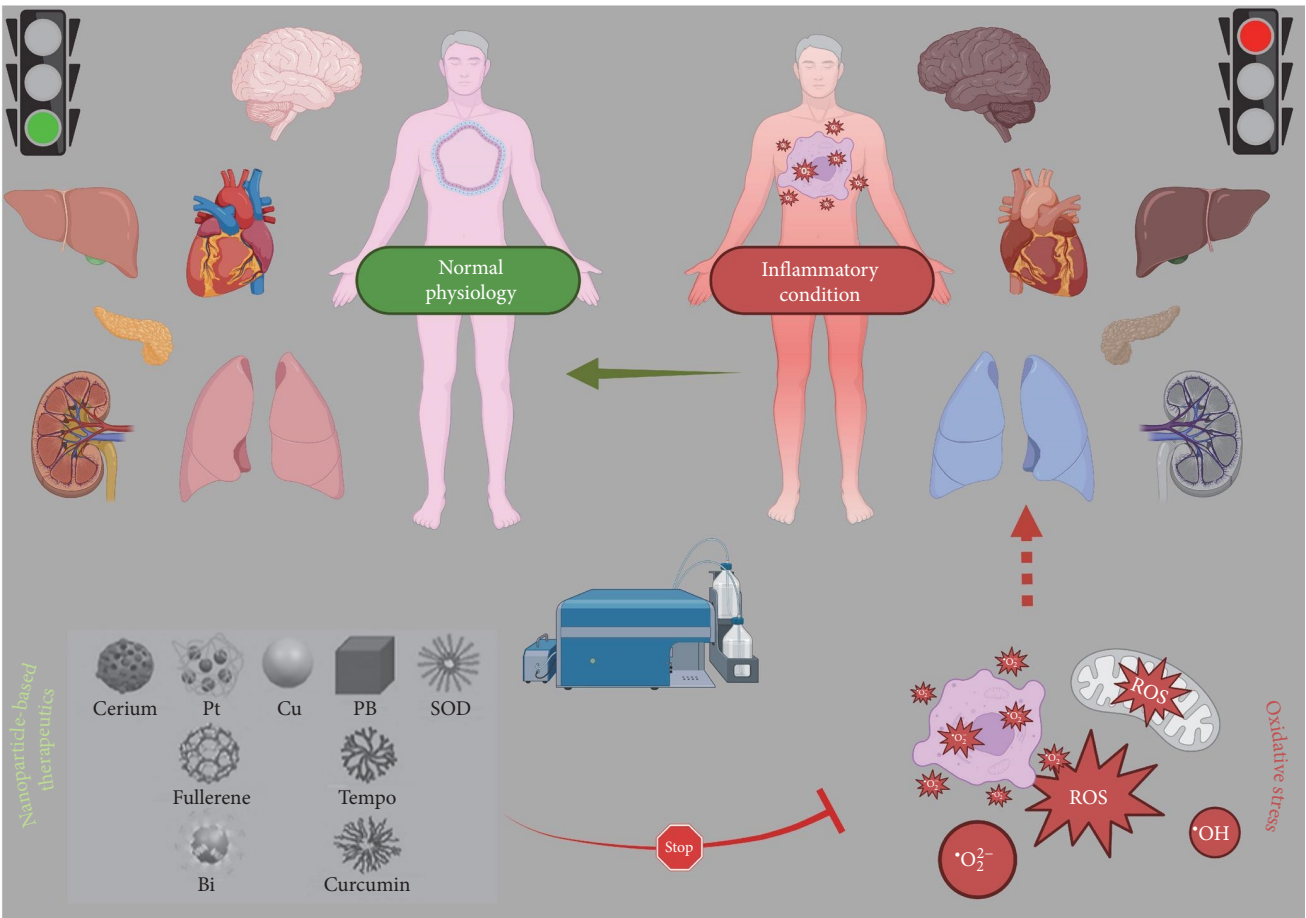
A schematic overview of the reactive oxygen species (ROS)-scavenging nanomaterials for resolving inflammation and acting as neuroprotective agents (created with BioRender.com).

One potential approach in the quest for neuroprotection involves utilizing nanoparticles that scavenge free radicals. Free radicals, together with reactive oxygen species (ROS) and reactive nitrogen species (RNS), have crucial impacts on neuroinflammation and oxidative stress, leading to harm to neurons [15–17]. NPs with intrinsic antioxidant properties or containing antioxidant chemicals are new approaches for alleviating oxidative stress and its associated effects [18]. Nanotechnology in medicine has given rise to the creation of nanoparticles that possess distinct physicochemical characteristics, making them well-suited for delivering drugs to specific targets and improving their effectiveness [19]. Nanoparticles with the ability to scavenge free radicals (Figure 1), such as those made from polymers or metal oxides, have demonstrated potential in preclinical research for their capacity to suppress ROS and RNS, thus reducing neuroinflammation and oxidative stress [19, 20].

In the last decade, the amalgamation of nanotechnology and artificial intelligence (AI) has drastically transformed neuroscience research. Different AI applications are improving our comprehension of intricate biological systems through advanced tools for analyzing data, recognizing patterns, and making close predictions [21]. In the field of neuroprotection, AI is playing a crucial role by enhancing the creation and refinement of nanoparticles, simplifying the procedures involved in drug development, and predicting prospective therapeutic outcomes [22]. Nevertheless, AI-powered analysis is essential for understanding the complex relationships between neuroinflammation, obesity, and nanoparticle treatments. Computational models have the ability to replicate biological interactions, forecast the behavior of nanoparticles inside a living organism, and determine the most favorable settings for neuroprotection [23]. It becomes easier to comprehend that the combination of AI with nanotechnology has the potential to expedite the process of translating from the laboratory to clinical applications.

### 1.1. Objectives

1. Summarize the ROS-scavenging efficacy of four nanoparticle classes (polymeric, liposomal, ceria, and quantum dots) in AD and PD models.
2. Detail state-of-the-art AI frameworks (GraphDRUG, automated deep learning [Auto-DL], etc.) used to optimize nanoparticle formulation and predict therapeutic outcomes.
3. Identify translational challenges and propose an integrated digital-twin pipeline for precision neuroprotective nanomedicine.

## 2. Neuroinflammation and Obesity Provide Dual Challenges

**Figure 2:**
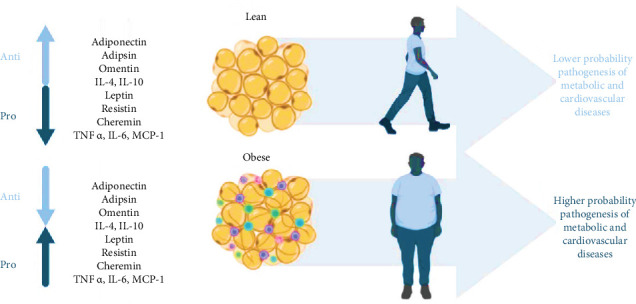
Adipokine dynamics correlated with an individual's adipose tissue condition. In a healthy adipose environment, proinflammatory adipokines decrease, and anti-inflammatory adipokines increase. Conversely, in obese adipose tissue, the pattern reverses, elevating the risk of metabolic and cardiovascular diseases (CVDs; reproduced from the open-access article by Clemente-Suárez et al. [25]).

The interface between obesity and neuroinflammation goes beyond a simple connection and has a substantial impact on both the overall health of the body and the central nervous system [3]. Neuroinflammation, formerly thought to be a response limited to the CNS, is now understood to be a dynamic process that is affected by external stimuli [5, 24]. Obesity has been identified as a powerful regulator of this process. Within the context of obesity, adipose tissue functions as an endocrine organ, releasing adipokines and proinflammatory cytokines [25, 26] (Figure 2). These signaling molecules can cross the blood–brain barrier and trigger inflammatory processes within the central nervous system [27]. Neuroinflammation is characterized by the activation of microglia, which occurs in response to these signals [27]. This activation results in the production of inflammatory mediators, hence continuing a cycle of inflammation. Moreover, alterations in the makeup of the gut microbiota caused by obesity play a role in promoting inflammation throughout the body, impacting the connection between the gut and the brain and regulating neuronal activity [28]. The reciprocal contact between the gastrointestinal tract and the central nervous system intensifies the influence of obesity on neuroinflammation, establishing a dynamic and interconnected network [29]. Studies in animals have revealed a link between the gut microbiota and various physiological changes, including stimulation of the vagus nerve. This connection influences subsequent effects on the brain and behavior [29, 30].

**Figure 3:**
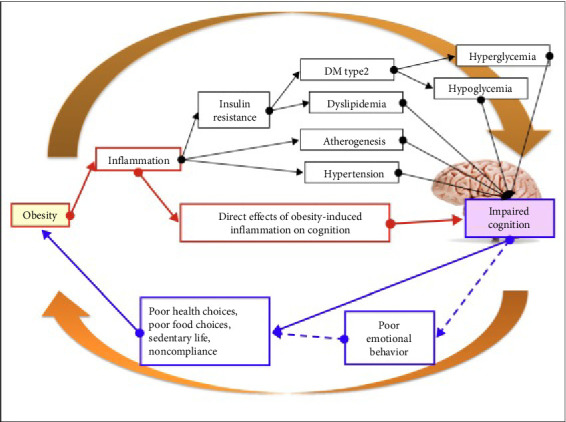
Schematic diagram of the progression of cognitive impairment in obese individuals stemming from chronic low-grade inflammation. This impairment can arise directly or indirectly through inflammation-induced metabolic disturbances (reproduced from the open-access article by Spyridaki et al. [35]).

The consequences of the connection between neuroinflammation and obesity include wide-ranging impacts on neurological well-being and cognitive abilities [29, 30]. Chronic low-grade inflammation, which is a common feature of both disorders, poses a risk to the structure and function of neurons [31]. Neuroinflammation plays a role in worsening the progression of neurodegenerative disorders such as Alzheimer's and Parkinson's disease [5–7, 32, 33]. Neuroinflammation caused by obesity has been associated with cognitive deficits and a greater likelihood of acquiring illnesses such as moderate cognitive impairment (MCI) and dementia [9]. Inflammation inhibits the ability of synapses to change and adapt, impairs the transmission of signals between neurons, and plays a role in the buildup of abnormal proteins in the brain [34]. Elucidating the complex connections between neuroinflammation and obesity is crucial for the development of successful approaches to safeguarding cognitive function and hindering neurodegeneration (Figure 3).

To effectively treat both neuroinflammation and obesity, it is crucial to have a thorough understanding of the current therapeutic strategies. The present techniques include lifestyle modifications, pharmaceutical substances, and anti-inflammatory treatments [9, 36]. Modifying one's lifestyle, including making changes to one's nutritional status and engaging in physical activity, is crucial for reducing inflammation related to obesity and is considered fundamental for treating neuroinflammation [9, 37]. Pharmacological strategies that specifically address inflammation, such as nonsteroidal anti-inflammatory medications (NSAIDs) and therapies that target cytokines, have demonstrated potential in preclinical research [31, 38]. Nevertheless, the successful application of these findings in clinical practice is still a difficult task due to concerns regarding long-term safety and potential unintended effects. This developing field of nanomedicine has led to the development of novel treatment methods, such as nanoparticles that can scavenge free radicals [39]. NPs specifically engineered to suppress oxidative stress and regulate inflammation have great promise to address both neuroinflammation and obesity simultaneously [40]. However, such a challenge has to overcome various obstacles, including the stability of nanoparticles, their in vivo compatibility with the biological system, and precise targeting for efficacy in a clinical context. Despite these breakthroughs in therapy, there are still limitations that exist. The diversity of neuroinflammatory reactions and the influence of obesity-related variables increase the complexity of treatment strategies [41]. Furthermore, the hurdles of establishing sustained treatment advantages include potential side effects, patient compliance with lifestyle alterations, and the necessity for long-term interventions. However, the complex connection between neuroinflammation and obesity highlights the necessity for comprehensive therapeutic strategies [9, 42]. Although current solutions display potential, the emergence of novel methods, specifically the utilization of nanoparticles that scavenge free radicals, has the ability to effectively address this dual dilemma.

## 3. Free Radical-Scavenging Nanoparticles: Mechanisms and Types

The fundamental strategy for protecting against neuroinflammation and obesity involves the use of cutting-edge free radical-scavenging nanoparticles [43, 44]. The core idea revolves around the interception and neutralization of ROS and RNS, which are commonly referred to as free radicals. These extremely reactive chemicals, which are involved in oxidative stress and inflammation, play a significant role in causing damage to neurons and are important factors in the development of neurodegenerative diseases [45, 46]. Nanoparticles with the ability to scavenge free radicals utilize their distinct physicochemical characteristics to combat oxidative stress [47]. These nanoparticles serve as powerful protectors of cellular components, safeguarding neurons and reducing the harmful consequences of neuroinflammation, either by directly interacting with free radicals or by releasing encapsulated antioxidants [47, 48]. Neuroprotective nanoparticles are characterized by a wide range of types, each possessing unique features that have been carefully engineered for successful intervention [49]. Polymeric nanoparticles, liposomes, metal oxide nanoparticles, and quantum dots have been identified as significant platforms for preclinical investigations [50, 51].

### 3.1. Polymeric Nanoparticles

Polymeric nanoparticles, derived from biocompatible polymers, excel in their capacity to provide precise release of antioxidants [52]. Some examples of nanoparticles are polylactic-co-glycolic acid (PLGA) and nanoparticles based on chitosan [19]. These formulations not only guarantee the continuous release of therapeutic substances but also demonstrate compatibility with biological systems, which is an essential factor for achieving successful neuroprotection [53–55].

PLGA nanoparticles have been widely researched for brain targeting of antioxidants. For example, curcumin-loaded PLGA-PEG NPs, intranasally administered at 25 mg/kg/day, once per week for 12 weeks, lowered hippocampal β-amyloid formation and deposition and tau hyperphosphorylation in an Alzheimer's mouse model, enhancing cognitive scores [56, 57]. Also, ferulic acid-encapsulated PLGA NPs reduced oxidative neuronal injury and restored hippocampal SOD content in a rat model of vascular dementia [58, 59]. A review affirmed that PLGA vectors improve brain retention and antioxidant activity across neurodegenerative disease models [52].

Especially when ionic gelation is used, the chitosan nanoparticles can be given by the nose or mouth in a way that sticks to mucus. In a recent study on rats with diabetes and obesity, it was found that giving 20 mg/kg of chitosan nanoparticles loaded with syringic acid orally every day for 21 days reduced glycation, oxidative stress, and neuroinflammation [60]. Furthermore, anthocyanin-rich extract (*Rubus fruticosus*) loaded chitosan NPs, administered at the dose of 10 mg/kg intranasally in a ketamine-induced schizophrenia rat model, enhanced catalase and GSH content of the brain, reduced TNF-α, and enhanced cognitive behavior [61]. Each one shows how polymeric carriers enhance antioxidant delivery through the BBB, provide for sustained release, and induce measurable in vivo neuroprotective effects.

### 3.2. Liposomal Formulations

Liposomes, based on their composition of a lipid bilayer, allow for flexibility in the encapsulation of antioxidants and the delivery of drugs at precise release points [62, 63]. The amphiphilic nature of these materials allows them to encapsulate both hydrophilic and lipophilic molecules, thus enhancing their flexibility as carriers in a wide range of medicinal treatments [62, 64]. Liposomes are likely to be effective carriers in precision medicine strategies for neuroprotection [65]. Liposomal curcumin shows enhanced delivery to the brain and antioxidant activity. In a mouse model of MPTP-induced Parkinson's disease, curcumin liposomes 15 mg/kg, single i.v. dose with polysorbate-80 coating) bypassed the blood–brain barrier and significantly attenuated α-synuclein aggregation and oxidative indicators in the striatum over free curcumin [66]. Liposomal coenzyme Q_10_ (CoQ10) has been found to have high neuroprotective activities in vivo. In a 21-day rotenone-induced PD model in mice, liposomal CoQ10 (2 mg/kg i.p. daily) greatly elevated SOD, CAT, and GSH values (*p*  < 0.0001), decreased TNF-α and IL-6, maintained dopaminergic neurons, and enhanced motor and cognitive function compared to free CoQ10 controls [67].

### 3.3. Metal-Oxide Nanoparticles

Titanium dioxide and cerium oxide are metal oxide nanoparticles that also have intrinsic antioxidant properties [68]. These nanoparticles, which are intended to scavenge free radicals, have been shown to be effective in protecting the nervous system in various experimental types [69, 70]. Metal oxide nanoparticles have unique features and could potentially modulate oxidative stress. Hence, they hold enormous potential as promising alternatives for therapies against neuroinflammation and the ramifications linked to obesity [71]. CeO_2_ NPs have exceptional regenerative antioxidant activities, undergoing Ce^3+^/Ce^4+^ cycling and behaving like superoxide dismutase and catalase enzymes [72]. Citrate-EDTA-stabilized CeO_2_ NPs (30 nm) administered by stereotactic brain perfusion in AD-model mice decreased amyloid-β deposition, microgliosis (↓ Iba-1), astrocytosis (↓ GFAP), and lipid peroxidation (↓ 4-HNE) following a single dose of 0.1 mM [72]. In metabolic disease, intraperitoneal administration of 0.5 mg/kg nanoceria twice a week for 6 weeks in obese Wistar rats markedly inhibited weight gain, decreased triglyceride production and leptin/insulin levels, and diminished body oxidative stress [73, 74].

Though explored mainly for toxicity, low-dose TiO_2_ NPs have exhibited systemic antioxidant functions under controlled inhalation exposure. In a continuous 7-week inhalation mouse study, 0.13 mg/m^3^ TiO_2_ NPs elevated brain glutathione by 32% and regulated systemic immune markers in favor of potential neuroprotection at sub-toxic environmental exposure [75]. Yet, increased or chronic exposure initiates neuroinflammation and disrupts learning, indicating a narrow therapeutic margin.

### 3.4. Quantum Dots

Quantum dots are nanocrystalline semiconductors with unique optical properties that have been employed for their inherent ability to deliver neuroprotection [76, 77]. When modified with antioxidants, quantum dots have an added advantage of offering both site-specific delivery and a significant enhancement in cellular uptake [78, 79]. This adaptability qualifies quantum dots as desirable agents for potent neuroprotection [79]. Carbon quantum dots (CQDs), particularly citric acid-based CQDs, have intrinsic antioxidant properties and neuroprotective activities. In PD animal models like *C. elegans* and mice, citric-acid CQDs suppressed paraquat-induced dopaminergic neuron loss, enhancing motor ability. The in vivo dosing up to 5 mg/kg p.o. Every 3 days for 12 days was found to be nontoxic [80]. Graphene quantum dots (GQDs) have shown α-synuclein antiaggregation and neuroprotection. Intraperitoneal GQDs (50 μg twice a week, 6 months) markedly decreased α-synucleinopathy, maintained dopaminergic neurons, and enhanced motor behavior in a mouse model of PD; they easily crossed the BBB in vivo [81]. Se-doped carbon nitride quantum dots (SegCN-QDs) applied in a PD rodent model (inhaled or injected at 5 mg/kg every third day for a total of 4 times) selectively inhibited microglial activation and enhanced α-synuclein clearance, lowering ROS and enhancing motor tests (*p* < 0.01) [80]. Quercetin-loaded CQDs (QECQDs) intrathecally injected (200 ng/g, once) in a mouse intracerebral hemorrhage model maintained cerebral blood flow, reduced ROS and MDA contents, alleviated neuron apoptosis (↓ cleaved caspase-3), and enhanced neurological scores after injury [82].

Each class of nanomaterial provides different advantages on the aspects of stability, biocompatibility, and controlled release kinetics. The selection of a specific type of nanoparticle is critical and is intrinsically linked to the complexity of the targeted application. A deep understanding of the specific features of each class is of great importance since this will offer researchers the much-needed leeway in the deliberate development of therapies meant to protect the nervous system [83]. Research on the potency of free radical scavenging nanoparticles is an essential aspect necessary in resolving the interconnected problems of neuroinflammation and obesity [84]. These studies provide essential information regarding the mechanisms underlying the action and can point to the potential of such nanoparticles as promising medicines in neuroprotection.

**Figure 4:**
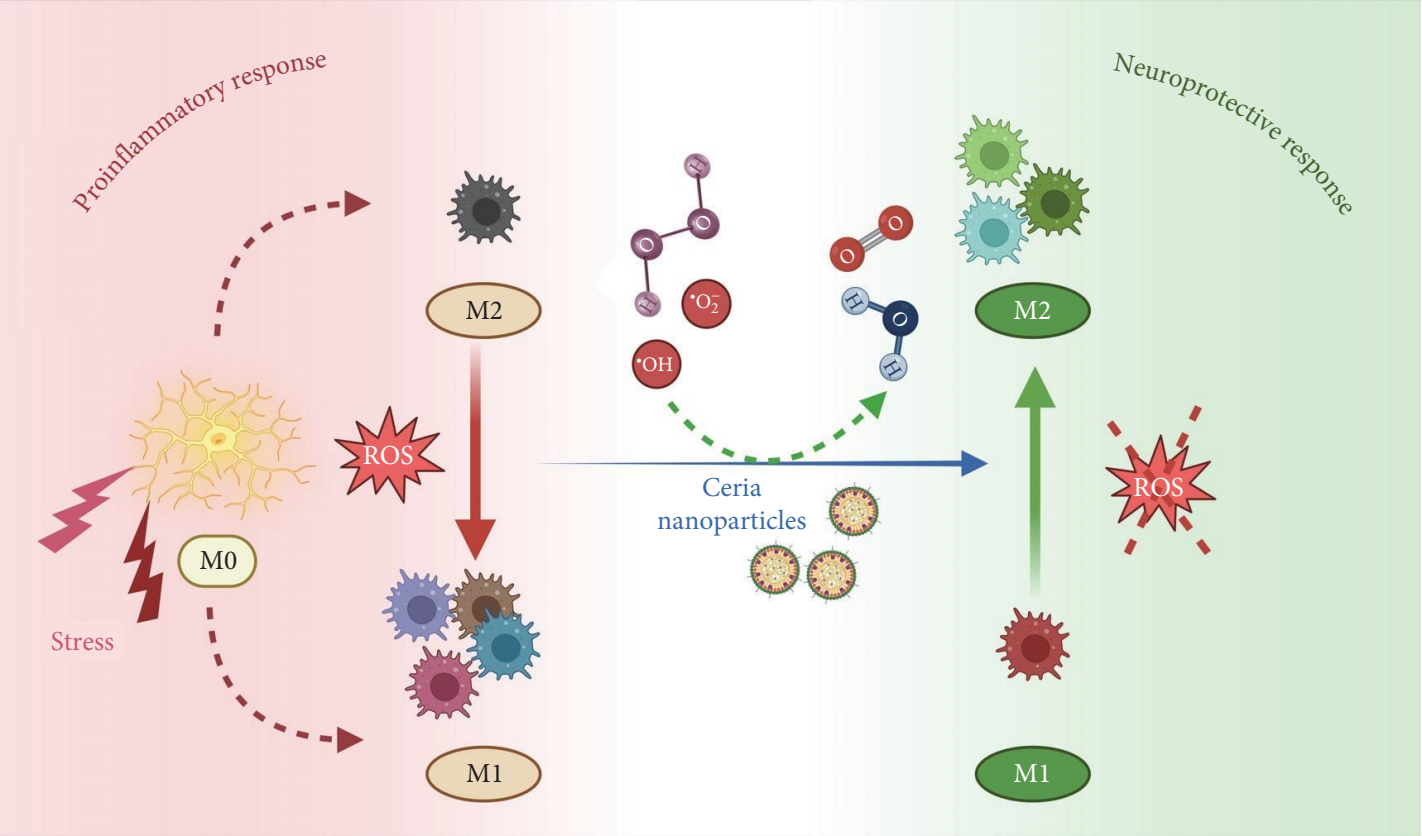
High-resolution schematic of ceria NP polarization and ROS scavenging in microglia. Ceria nanoparticles induce a shift in microglial polarization, transitioning from a proinflammatory M1 phenotype to an anti-inflammatory M2 phenotype. This transformation is facilitated by the scavenging of various reactive oxygen species (ROS) that are overproduced in response to stress stimuli (created with BioRender.com).

Various studies have successfully shown the high efficiency of nanoparticles in scavenging free radicals to address the intricacies related to neuroinflammation and the effects associated with obesity [85, 86]. These experiments are encouraging and have variously shown the mechanisms and the potential therapeutic application of different types of nanoparticles. Among those, studies related to the use of polymeric nanoparticles have utilized PLGA very effectively [87]. NPs, having a high concentration of antioxidants, can deliver their contents over an extended period of time and make their contents more available for biological processes [88, 89]. The result is an improved efficacy in neuroinflammation treatment, hence providing a promising way for therapy that is aimed at preserving neural health. Liposomes, which have been considered for their efficient encapsulation of antioxidants, are a promising approach for neuroprotection [90]. Some promising results were achieved from the use of liposomes as an antioxidant delivery system, for example, curcumin or resveratrol. Some formulations have been found to be effective in lowering neuroinflammation and reducing oxidative stress marker expression in experimental studies [85, 91]. This would create an opportunity for using liposomal approaches to mitigate the effects of neuroinflammatory processes. Some metal oxide nanoparticles, like cerium oxide, have also come into focus lately due to their intrinsic antioxidant activity [68]. Nanoscale cerium oxide, with its design specifically to neutralize harmful free radicals, presents neuroprotective properties in a variety of models for neurodegenerative diseases (Figure 4). These findings highlight the potential of metal oxide nanoparticles as strong candidates in the field of neuroprotection, especially for reducing oxidative stress in the central nervous system [92]. Antioxidants, which are nanoparticles made of semiconductors and possess distinctive optical characteristics, have been deliberately included in quantum dots. This alteration endows quantum dots with versatility, allowing precise delivery with improved absorption by cells. The characteristics of quantum dots make them highly adaptable instruments in the range of tactics used for neuroprotection. This highlights their ability to effectively address the complex issues associated with neuroinflammation and the damage caused by obesity [93].

It is evident that free radical-scavenging nanoparticles are amenable to meeting the challenges of neuroinflammation and obesity at the same time and have generated a vast and developing body of literature. Multiple approaches by various nanoparticle formulations contribute to the building body of evidence of their importance as potential agents in the area of neuroprotection [94]. We observed in the key research studies reviewed in this paper that a greater appreciation of the intricate relationship between nanoparticles and neuro-health emerged, which implies hope for new and effective therapeutic treatments.

## 4. AI Integration in Neuroprotection

**Figure 5:**
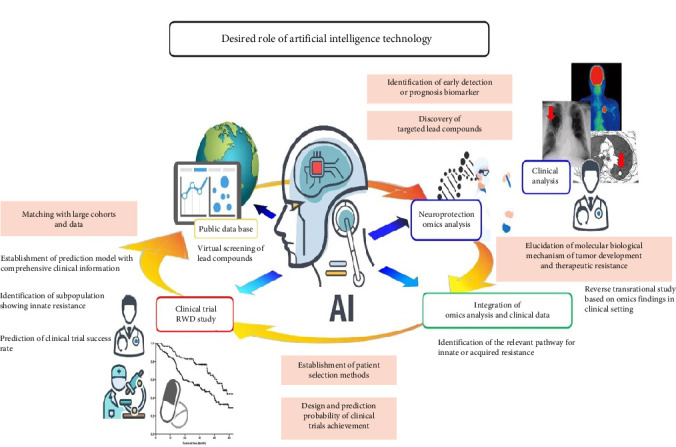
Workflow of AI-driven nanoparticle design: (1) data curation, (2) model training, (3) virtual screening, and (4) experimental validation. The future direction and potential role of AI in shaping new treatment strategies are poised to be transformative, as can be observed in this figure. AI holds the promise of revolutionizing various aspects of healthcare and medicine, offering innovative approaches to the development of treatment strategies (reproduced from the open-access article by Tanaka et al. [99]).

The approach toward integrating AI and neuroscience has proven very promising (Figure 5) as it may revolutionize our attempts toward effective neuroprotection [95]. AI is such a powerful computational tool that it also surpasses established methods in restructuring our understanding of complex neurological systems and in advancing individually tailored medical treatments and strategies [96, 97]. The influence of AI within neuroscience includes diverse sophisticated methodologies, including machine learning, deep learning, and natural language processing [95]. Machine learning algorithms, well-taught on large datasets, will uncover complex patterns in neurological data to reveal insights that cannot be captured by standard analytical methods [95]. The ability to analyze complex information has to be understood to know the intricate relationships among the different elements that affect neuroprotection. Deep learning, which is influenced by the complex structure of neural networks, allows for an unprecedented level of research in neuroscience [98]. This methodology surpasses the constraints of conventional analytical methods, allowing for the detection of minute yet essential patterns that underpin neuroprotection systems (Figure 5).

AI has brought about profound transformations within neuroprotection, especially in the progress of precision medicine [100]. Tailored therapy interventions based on AI-produced knowledge open a new dimension where no intervention is collective but rather very tailor-made to apply or suit the individual characteristics of each patient [101, 102]. This individualized strategy comes with major implications in the approach for dealing with both neuroinflammation issues and effects related to obesity. Machine learning techniques are utilized to identify complex patterns within neuroimaging and chemical data [103]. AI-driven studies efficiently unravel intricate connections and provide prediction models for understanding interactions between free radical-scavenging nanoparticles and the neurological system [104]. This feature significantly accelerates the design process as well as optimization of nanoparticles to ensure maximum effectiveness in neuroprotection. Deep learning technique is a crucial step of extracting hierarchical features, which is significant in deeply understanding the intricate relationship between neuroinflammation and the effects of obesity [9, 105]. Deep learning analyses provide important insights to researchers, allowing them to find even subtle correlations within large datasets and uncover hidden inter-relationships [106]. Discoveries such as these not only enhance our understanding of neuroprotection but also allow us in the development of specific therapies. The use of AI in neuroprotection is a step beyond mere technological innovation, but rather the key toward the future when treatments are well matched to the complexities of individual neurobiology [107]. That being said, it is relevant when cross-comparing the fields of AI and neurology to discuss the potential clinical implications of free radical scavenging nanoparticles. Nevertheless, it is also crucial to cogitate, discuss, and analyze the potential of such materials for medical applications and perspectives for clinical setting practice.

### 4.1. Utilizing AI in Developing Advanced Free Radical-Scavenging Nanoparticles for Neuroprotection

The field of neuroprotection is evolving, and the strategic design and careful consideration of free radical-scavenging nanoparticles would significantly require the role of AI. Advanced artificial techniques are required for the effective comprehension and manipulation of the complex relationships between the characteristics of the nanoparticles and the biological system and therapeutic intervention results [108]. AI plays a major role in designing and tailoring nanoparticles for better therapeutic qualities [108] and, hence, significantly contributes to neuroprotection. Algorithms run by massive databases that contain extensive data about the properties of nanoparticles, drug release rate, and biological interactions collaborate to orchestrate a very accurate and precise process [109]. This symphony will allow scientists to predict the best nanoparticle composition so as to ensure maximum efficacy against free radicals, and thus deliver robust neuroprotection. Importantly, the iterative nature that AI-driven design has endowed on the development of novel nanoparticles that not only accelerates the process but also improves their therapeutic potential to a great level [110]. Through the analysis of intricate patterns in intricate datasets, AI enables researchers to customize nanoparticles to meet the precise requirements of neuroprotection, a task that cannot be achieved using conventional methods.

AI-based pharmacokinetic models act as a reference or guide for the complex scavenging path of free radicals through the body by nanoparticles [111, 112]. It does more than the traditional techniques as it encompasses biodistribution, metabolism, and excretion. The result is an in-depth understanding of pharmacokinetics in these nanoparticles, which allows for useful insights that dictate their journey from administration to function at the target site. The accuracy of AI in pharmacokinetic modeling is critical for predicting therapeutic concentrations in defined organs [113]. This discovery gives researchers a “beacon” guiding treatment protocols toward enhancement and reiterating that maximal exploitation of the therapeutic efficacy of free radical-scavenging nanoparticles is achieved. AI algorithms are vital for enhancing systems of targeted drug delivery, thus giving birth to personalized precision medicine [114]. Personal data, such as genetic profiles and features of illness, are used in AI to enable the synthesis of nanoparticles that can accurately target afflicted areas of the brain [115]. The strategy is individualized. Not only does it reduce unintended effects, but therapeutic effectiveness is also more improved through applying neuroprotective techniques uniquely tailored to an individual's biological characteristics. The integration of AI and targeted medication delivery can be considered as a transition from a generalized intervention to more customized therapies, wherein the nanoparticles act as a specialized carrier that meets individual requirements on a case-by-case basis [108, 115]. Using this insight of AI, this refined strategy would be able to modify the neurological approach to better efficacy and reduce adverse effects. In essence, the diverse contributions of AI to the design of nanoparticles, pharmacokinetic modeling, and drug delivery to specific targets represent exciting advances in the quest for effective neuroprotection [108, 113].

### 4.2. AI-Driven Insights and Advancements in Neuroprotection

Innovative applications have revolutionary promise for the future of nanoparticle-based neuroprotection [116]. The beneficial connection between AI and neuroprotection has been demonstrated by these breakthroughs and surprising insights. AI-powered predictive models are becoming valuable tools for understanding the intricate relationship between nanoparticle efficacy and neuroprotection [117]. Through analytical examination of various datasets, these models identify complex relationships between nanoparticle characteristics and therapeutic results. This ability enables researchers to anticipate the effectiveness of particular nanoparticle compositions in removing harmful free radicals and reducing inflammation in the brain [20]. Predictive modeling not only allows for foresight but also efficiently assists researchers in identifying promising candidates for further examination. This strategy not only simplifies the research process but also places neuroprotective efforts on a path characterized by effectiveness and focused examination of the most promising areas. AI algorithms, which are smoothly incorporated into imaging systems, are leading the way in a new era of instant monitoring for neuroinflammation [118]. NPs, which are equipped with imaging agents and responsive to inflammatory indicators, can serve as active monitors to continuously track inflammation levels in complex neuronal environments [119, 120]. AI analyzes a large amount of imaging data, offering both qualitative and quantitative evaluations that enable researchers to make well-informed conclusions immediately. The utilization of AI enables a dynamic monitoring capability that surpasses the constraints of conventional static assessments [121]. This approach enables the prompt adjustment of neuroprotection measures according to the changing dynamics of neuroinflammation. The outcome is a fundamental change in approach from responding to situations after they occur to taking proactive, flexible actions that are in line with the immediate requirements of the brain environment.

AI plays a vital role in propelling innovation in neuroprotection as it enables drug repurposing [122]. The AI systems systematically analyze huge databases of drugs and molecular interactions, suggesting future candidates for repurposing for neuroprotection [113, 123]. With this strategic approach, research into substances with established safety profiles is catalyzed and redirected to reduce neuroinflammation once those substances are encapsulated within nanoparticles. The application of AI in drug repurposing accelerates the development of neuroprotective drugs and fully exploits the valuable information embedded in well-established pharmacopoeias [22]. It aligns with the principles of sustainability in pharmaceutical sciences, saving time for the performance of long clinical trials while using the pharmaceutical potential of compounds previously proven to be safe and effective in other contexts [124]. AI-driven insights provide us with an all-inclusive toolkit that can propel the industry to unprecedented heights, encompassing accurate nanoparticle creation, real-time monitoring, and innovative drug repurposing [125].

Aside from nanocarrier screening, AI has led to neuroprotective small molecules that were tested in animal models. Tu et al. [126] employed large-language and graph neural network models to discover the RIPK3 inhibitor HG9-91-01, which, when it was delivered systemically in a model of acute glaucoma in mice, protected retinal ganglion cells and lowered necroptosis markers. Romeo-Guitart et al. [127] used the TPMS systems-biology AI platform to rodent nerve-root avulsion proteomic data and identified the drug combination NeuroHeal (acamprosate + ribavirin). Intrathecal administration of NeuroHeal for 20 days after injury enhanced motoneuron survival by 45 % and locomotor recovery [127].

### 4.3. State-of-the-Art AI Frameworks for Neuro-Nanomedicine

Recent advances in AI-based molecular modeling and nanomaterial design are assisting in a wide range of applications in neuro-nanomedicine. Auto-DL employs Bayesian optimization and reinforcement learning to rapidly optimize nanoparticle parameters such as polymer composition, antioxidant loading, and surface modifications. Zheng et al. [128] have explained that Auto-DL reduced the optimization cycle and significantly accelerated the discovery of effective neuroprotective nanoformulations. For instance, the AlphaFold-Multimer [129]. This new version of AlphaFold predicts protein–protein and protein–ligand complex structures with good accuracy by modeling multimeric complexes and small molecule binding. In neuro-nanomedicine, it facilitates structural modeling of protein–NP interfaces to support rational surface engineering to improve BBB targeting and minimize immunogenicity [130]. GraphDRUG employs graph-based deep learning for the prediction of pharmacokinetic and pharmacodynamic properties of nanoformulations. Graphically representing nanoparticle properties (e.g., size, surface charge, hydrophobicity) and biological interactions, GraphDRUG has optimized nanoparticle design, enhancing formulation accuracy through speedy screening of multiple NP combinations for neuroprotective efficacy [131]. GPT-Chem, a version of GPT-based transformer architectures, produces new chemical structures designed for antioxidant-loaded nanoparticle formulations. Applying structure–activity relationship (SAR) information, GPT-Chem can suggest new antioxidants and biocompatible polymer carriers, with recent examples showing about 30% of generated compounds passed stringent computational ADME-toxicity prediction, proceeding to experimental validation stages [132]. Collectively, such AI models have the potential to advance neuro-nanomedicine through enabling quick, accurate, and experimentally verified design routes. They save experiment time, simplify the development of targeted neuroprotective nanoparticles, and enable the translation of nanoparticle-based therapies from the bench to the clinic.

#### 4.3.1. Neuroinflammation Simulations Using AI

New AI-powered computational models have now been constructed to model how nanoparticle formulations control neuroinflammatory cytokines such as IL-1β and TNF-α. For instance, physics-informed neural networks (PINNs) incorporate the embedded underlying physical laws (e.g., diffusion, interstitial transport, kinetics of reaction) into the training of neural models themselves. Though mainly used in engineering (e.g., thermal fields, polymer systems) [133], this approach can be taken in neuro-nanomedicine. Wei et al. [134] trained ensemble models (gradient boosting, random forest) on a database of >200 nanozyme formulations such as Fe_3_O_4_, CeO_2_, and MnO_2_ to estimate catalytic parameters (*k*_at_, *K*_m_) for H_2_O_2_ decomposition. Their explainable-ML workflow obtained *R*^2^ = 0.92 for the prediction of *k*_at_; SHAP analysis identified surface defect density and dopant fraction as the leading determinants of activity. Three high-activity Fe_3_O_4_ nanozymes identified by the model were prepared and exhibited H_2_O_2_ decomposition rates to within 10 % of ML predictions by UV–vis assay [134]. Zhuang et al. [135] combined a graph convolutional network encoding atomic-scale characteristics (doping ratio, facet index, coordination number) with COMSOL finite-element simulations of nanozyme diffusion and reaction in a 3D tissue-mimetic hydrogel. This hybrid model forecasted ROS scavenging rate and spatial pattern with 80 % accuracy compared to fluorescent reporter imaging. In an LPS-induced neuroinflammation mouse model (Fe_3_O_4_ nanozyme, 0.5 mg/kg i.p.), it predicted a 55 % hippocampal decrease in IL-1β, in agreement with ELISA within 12 % error [135]. Liu et al. [136] designed a Pt@PCN222-Mn cascade nanozyme and employed COMSOL reaction–diffusion modeling to model stepwise ROS conversion in inflamed brain tissue. Their in silico model was able to predict > 70 % reduction in H_2_O_2_ and superoxide, and 50 % reduction in IL-1β and TNF-α at 0.2 mg/kg i.v. in a traumatic-brain-injury mouse, subsequently verified by in vivo ELISA and immunohistochemistry [136]. These AI models, therefore, transcend outcome-level effects and provide a mechanistic explanation of nanoparticle interaction with ion channels and neural firing dynamics.

#### 4.3.2. NP–Neuronal Interaction AI Modeling

In the study by Busse et al. [137], they integrated whole-cell patch-clamp recordings of cAgNP-exposed chromaffin cells with Hodgkin–Huxley (HH) modeling that was fitted using a Differential-Evolution algorithm. They determined NP-induced parameter shifts in sodium-current amplitude and gating kinetics, and then injected these into a thalamocortical network model to forecast changes in neuronal firing patterns and circuit rhythms. Furthermore, Martini et al. [138] developed the neuronal spike shapes (NSSs) approach. This way transforms action-potential waveforms into triangular feature vectors and applies unsupervised clustering plus downstream differential-expression analysis. When applied to patch-clamp data, NSS correctly discriminates fast-spiking versus regular-spiking excitability states by metrics like spike width and afterhyperpolarization. Also, NSS is known to reveal changes in NP-treated cultures that lead to broader spikes and longer repolarization phases. This is a direct link between NP membrane contacts and the altered excitability profiles. Hernandes et al. [139] also showed a gradient-boosted decision-tree pipeline (called autoMEA) that can find the best burst-detection parameters for 24-well MEA datasets. Evaluated on primary hippocampal neurons, autoMEA equaled expert-level accuracy in identifying simple and reverberating bursts. Used on NP-exposed neuronal networks, it is able to measure changes in burst frequency, synchronization, and reverberation dynamics, offering a scalable assay of NP-induced network modulation

## 5. Translational Potential and Clinical Perspectives

The transition from lab findings to practical applications in clinical treatments is the final stage of development for any neuroprotective approaches. This is especially true for those of the latest emerging technology of nanoparticles that scavenge free radicals. Nanoparticles stand out as a promising candidate to deal with the heavy complexity of neuroinflammation and the effect of obesity in the clinical scenario [40, 140]. The specially designed free radical scavenging nanoparticles, developed with the combined power of AI through detailed developments and enhancements, yield highly potent platforms ready for practical application [141]. These nanoparticles may decrease neuroinflammation and alleviate the impact of obesity on neurological health. The integration of high technology with scientific ingenuity provides a soft platform with significant implications for future neuroprotection [142]. More importantly, the different translation importance must be considered. This means careful attention to the existing clinical trials that are currently proceeding in the clinical stage. These experiments challenge the efficacy and safety of free radical-scavenging nanoparticles under real-world applications, thereby enabling researchers to obtain essential information regarding efficacy and safety. The narrative further extends to include the challenges that cannot be avoided at every step in this process of translation, along with the predictability and control of future approaches in clinical applications. This thorough review covers not only the existing situation but also puts us at the cutting edge of understanding the therapeutic potentialities of free-radical scavenging nanoparticles. This future envisions a smooth and seamless transition from scientific theories to relevant ground-breaking changes in neurological welfare.

### 5.1. Examination of Clinical Trials and Their Outcomes

What we understand as of now is that the progression from preclinical promise to clinical reality takes on a prime phase in the form of conducting active clinical trials. These are essentially intense proof grounds for testing the efficacy and safety of free radical-scavenging nanoparticles in the ever-changing landscape of the human patient [143]. The inquiry includes considerations that all serve to combine to shape the process of translating research results into real-world applications. These factors include careful planning of experiments, various types of nanoparticle usage, and critical assessment of the results. The careful and precise design of these clinical trials represents the first stage in the process of translating research findings into practical applications [144]. The design of a future experiment must take into account the many factors involved with nanoparticle formulation, dosing, and delivery routes, as these play a significant role in determining how the clinical investigation will follow. In this light, other types of nanoparticles come to the fore, taking on vastly different attributes that are now ready to be investigated. Of these, one finds polymeric nanoparticles, liposomes, metal oxide nanoparticles, and quantum dots. The variety of formulations developed for nanoparticles is extensive, demonstrating the scope in which researchers can utilize their work to translate their discoveries into the clinical setting, with each formulation demonstrating different potential within the therapeutic setting. At the heart of these clinical trials are specific, detailed analyzes of whether the treatment maintains neurological function [144]. Changes in cognitive functions, neuroinflammatory markers, and neurological sequelae related to obesity improvement are important clinical outcomes. The effect of nanoparticles in scavenging free radicals is under thorough investigation in animal models as researchers find clear evidence in real human patients regarding their ability to protect neurological health [20].

Apart from effectiveness, safety and tolerability also represent a critical aspect in the process of translation [145]. The adverse effects associated with such conditions should be closely monitored. Nanoparticle therapies should be critically evaluated in terms of their risk–benefit profile. The thrust in this regard of safety reflects the moral obligation to take precedence in the affairs of patients so that the putative benefits of neuroprotection will not be outweighed by unexpected risks. The translational process with the adoption of the principles of personalized medicine becomes more complex because the patient is stratified on the basis of the genetic, metabolic, and disease-specific characteristics. This method is intended to identify specific groups in various patient populations that may benefit most from neuroprotection due to nanoparticle use [145, 146]. With this approach, the practice of translation is no longer a general procedure but a personal plan that recognizes and makes available the unique biological features of every individual subject. Ultimately, the review of ongoing clinical trials and their findings is the interactive tango between scientific hypotheses and live clinical medicine. The journey includes a variety of components, including careful planning of trials, development of different nanoparticle formulations, effectiveness evaluation, safety assessment, and personalized medicine. Over the course of such trials, they not only provide an overview of the current state of clinical research but also help in advancing neuroprotective strategies. These strategies should introduce nanoparticles that scavenge free radicals into the clinical workflow, bringing real benefits to patients struggling with the complex issues involved with neuroinflammation and the neurological effects of obesity.

### 5.2. Challenges and Future Directions in Clinical Applications

Clinical application of free radical-scavenging nanoparticles from the laboratory is not without challenges [20, 147]. These areas, therefore, have to be successfully addressed for these approaches to be fully exploited in hospital settings. The ability of nanoparticles to target the desired brain region while maintaining appropriate distribution throughout the body is a very complex and intricate challenge. Additionally, the heterogeneity of patient anatomies and illness pathophysiologies complicates the attainment of optimal treatment doses in some neuronal regions in some cases [148]. Nevertheless, long-term follow-up studies are required to provide sustained efficacy while establishing the long-term safety of nanoparticle treatments. An intense clinical outcome needs to be assessed since the additive effects might vary, especially in conditions such as chronic neuroinflammation and obesity. Also, the presence of regulations and lack of standardization pose obstacles to implementing standardized procedures for the development and approval of neuroprotective treatments based on nanoparticles [143]. It is necessary that robust systems that ensure safety, efficacy, and consistent manufacturing processes get regulatory approval and broader clinical acceptance. That said, the convergence of cutting-edge neuroscience, AI, and therapeutic applications has raised ethical concerns and societal consequences [149]. Inclusion of those aspects, such as obtaining informed consent, ensuring equal access to advanced treatments, and responsibly sharing AI-derived insights, requires careful consideration.

It is also important to discuss the potential avenues for further exploration in this area of contemporary research and development. The first step would be to successfully address the obstacles that would offer a clear path for future advancements in the clinical use of nanoparticles that scavenge free radicals. For example, the combination of nanoparticle interventions with other neuroprotective techniques, such as lifestyle adjustments and pharmacotherapies, has the potential to improve overall effectiveness and target various aspects of neuroinflammation and the impacts connected to obesity [150]. The use of cutting-edge imaging technology enables live tracking of nanoparticle dispersions in the body and assessment of their therapeutic impact. Progress in imaging techniques has enhanced our understanding of the intricate dynamics of nanoparticle behavior in the human nervous system [151]. Future research should prioritize patient-centric outcomes, focusing on gains in quality of life, functional independence, and cognitive well-being, which are outcomes that directly impact patients [152]. Integrating patient-reported outcomes enhances the clinical course of action and guarantees that interventions are in line with the wider objectives of patient care.

Ultimately, the translational potential and clinical promise of free radical scavenger nanoparticles constitute an advanced field where scientific creativity meets constantly evolving realities of practice in patient care. The neuroprotective pathway depends on scientific sophistication coupled with the desire to translate findings into realizable gains in brain health, as clinical trials emerge, and researchers are confronted with challenges [153]. All these will converge together to build a bright future where nanoparticles that remove bad free radicals from the body are the main parts of the arsenal built to protect the nervous system.

## 6. Discussion

The interesting research into the complexities of neuroprotection, supported by the synergy of AI, free radical-scavenging nanoparticles, and neuroinflammation and obesity dual challenges, has not only resulted in the creation of a volume of new scientific evidence but also has endless translational potential [154]. Through this review article, we have described the connectivity of different areas of study, such as the precise fabrication of nanoparticles and the incorporation of AI-generated insights. This would help in the comprehension of the latest knowledge that has the potential to change how we approach neurological health as a society. The main discoveries from this investigational discussion highlight the exceptional adaptability of nanoparticles that eliminate free radicals for reducing neuroinflammation and combating the neurological effects of obesity. The spectrum of nanoparticle formulations, including polymeric nanoparticles capable of controlled release and metal oxide nanoparticles with inherent antioxidant properties, constitutes a highly sophisticated set of tools for neuroprotection [155]. The integration of AI into this field enhances accuracy, expanding from the use of predictive modeling to guide nanoparticle design to the use of real-time monitoring to enable adaptive therapies [113, 114, 156]. The core focus of this comprehensive review revolves around the seamless combination of neuroprotective capabilities, AI, and the intricate difficulties presented by neuroinflammation and obesity. This integration aims to create a future where interventions go beyond reacting to situations and instead prioritize adaptability as the standard. The future is characterized by precision medicine, which is customized to suit the specific characteristics of individual patients. The combination of neuroprotective capabilities and AI-driven insights not only revolutionizes conventional research approaches but also redefines the field of therapeutic treatment.

The efficacy assessments and safety evaluations conducted in future trials could provide valuable guidance for future research efforts aimed at achieving concrete advancements in neurological well-being. Researchers need to carefully analyze the effects of these nanoparticles in the clinical narrative, aiming to find evidence of both effectiveness and long-term safety, as well as insights into patient outcomes. Moreover, the integration of AI with nanoparticle design ushers in a novel era of precision medicine, where therapies are not only tailored for effectiveness but also infused with a deep comprehension of particular patient subtleties [100, 115]. Stratifying patients on the basis of genetic, metabolic, and disease-specific characteristics goes beyond theoretical concerns and is now seen as a practical technique to maximize benefits and minimize risks. The simultaneous occurrence of neuroinflammation and obesity, formerly considered separate issues, gives rise to a complex situation in which therapies aim to target the interrelated network of physiological processes. With the ability to scavenge free radicals, AI can be used to navigate this environment skillfully, providing a versatile way to mitigate the harmful impact on neurological well-being.

## 7. Conclusion

Accurate neuroprotection against obesity-associated neuroinflammation requires the coupling of ROS-scavenging nanoparticles with AI-guided design. Our review emphasizes four nanoparticle platforms, such as polymeric (PLGA, chitosan), liposomal, ceria-based metal oxides, and quantum dots, that have strong antioxidant and anti-inflammatory activities in Alzheimer's and Parkinson's rodent models. AI platforms like GraphDRUG, Auto-DL, and hybrid GNN–COMSOL simulations enhance formulation screening, optimize biointeractions, and forecast cytokine suppression and biodistribution. Mechanistic understanding from PINNs and cascade nanozyme models explains spatiotemporal ROS modulation, whereas electrophysiological AI interpretations decrypt nanoparticle–neuron interactions. Challenges remain in scalable production, BBB penetration, long-term biocompatibility, and regulatory concordance. Future progress must focus on multiomics integration, patient stratification, and adaptive clinical trials based on real-time AI monitoring. By integrating nanotechnology, computational modeling, and precision medicine, the discipline is on the verge of bringing precision antioxidant nanoparticles into efficacious and safe neurotherapies for metabolic and inflammatory brain diseases.

## Data Availability

The data are available upon request from the authors.
